# Self-compassion and psychological well-being in collegiate athletes: the mediating role of resilience and the moderating role of self-efficacy

**DOI:** 10.3389/fspor.2026.1814251

**Published:** 2026-06-17

**Authors:** Kyoo-Ho Lee, Kyung-Rok Oh

**Affiliations:** Department of Coaching, Kyung Hee University, Yongin, Republic of Korea

**Keywords:** collegiate athletes, psychological well-being, resilience, self-compassion, self-efficacy

## Abstract

**Introduction:**

This study examined how dimensions of self-compassion are associated with psychological well-being among collegiate athletes and whether these associations operate through resilience, contingent on levels of self-efficacy.

**Methods:**

A total of 342 Korean collegiate athletes completed validated measures of self-compassion, resilience, self-efficacy, and Ryff's multidimensional psychological well-being domains (autonomy, environmental mastery, personal growth, purpose in life, and positive relations with others). Conditional process analyses were conducted using PROCESS Macro Model 14.

**Results:**

The results indicated that resilience significantly mediated the relationships between multiple self-compassion dimensions and psychological well-being outcomes. Moreover, self-efficacy moderated several of these indirect pathways, such that the magnitude and direction of the mediated effects varied across well-being domains. Specifically, moderated mediation was supported for autonomy, environmental mastery, purpose in life, and positive relations with others. In contrast, no significant conditional indirect effects were observed for personal growth, although certain self-compassion dimensions exerted significant direct effects on this outcome.

**Discussion:**

Taken together, these findings elucidate the multidimensional psychological mechanisms through which self-compassion contributes to athletes' well-being and highlight the critical roles of resilience and self-efficacy in shaping mental health outcomes. The results underscore the value of interventions that simultaneously strengthen resilience and enhance self-efficacy to promote psychological well-being among collegiate athletes..

## 1 Introduction

Collegiate athletes are frequently exposed to elevated levels of stress arising from the dual demands of academic achievement and competitive sports participation. Managing these concurrent pressures often requires sustained psychological effort, and prolonged exposure to such demands may compromise psychological well-being and increase vulnerability to mental health concerns, including anxiety, burnout, and emotional dysregulation. In response to growing awareness of these challenges, recent research has increasingly emphasized the identification of psychological resources that help student-athletes maintain well-being and adapt effectively to stressors inherent in both academic and athletic contexts. Understanding how these resources operate is critical for advancing theory and informing interventions aimed at promoting mental health and optimal functioning among collegiate athletes.

### 1.1 Background and rationale

Collegiate athletes are frequently exposed to high-pressure environments that demand consistent physical performance, academic achievement, and emotional resilience (1, 2). In particular, South Korean student-athletes often encounter intensive training regimens and hierarchical coach–athlete dynamics shaped by collectivist cultural values, which may constrain perceived autonomy and elevate psychological stress (3, 4). These contextual challenges can undermine mental health and increase the risk of burnout, anxiety, and maladaptive coping strategies (5, 6). Accordingly, sport psychologists and sport organizations have increasingly emphasized the prioritization of athletes’ psychological well-being as a core component of sustainable performance and long-term development (7, 8). Beyond the scope of performance enhancement, clinical psychology perspectives underscore that safeguarding athletes’ mental health is a fundamental necessity in its own right. Clinical frameworks emphasize the critical need for early identification and targeted interventions to prevent severe psychopathology and clinical disorders—such as clinical depression and severe anxiety—which can be exacerbated by the chronic stressors inherent in elite sport environments (9, 10). Therefore, integrating clinical mental health considerations is essential for comprehensively protecting athletes’ overall psychological integrity. Psychological well-being—commonly defined through Ryff's (11) multidimensional framework—encompasses autonomy, environmental mastery, personal growth, purpose in life, and positive relations with others (11, 12). These dimensions represent eudaimonic aspects of optimal functioning and are particularly critical in competitive sport settings, where athletes must continuously regulate emotions, adapt to performance-related pressure, and sustain motivation (7, 8). Among the personal psychological resources that support well-being, self-compassion has received growing empirical attention (7). Conceptually, self-compassion reflects a balanced way of relating to oneself in times of difficulty and is characterized by compassionate self-responding (e.g., self-kindness and common humanity) as well as reduced self-critical tendencies (e.g., self-judgment and feelings of isolation) (13, 14).

Among athletes, higher levels of adaptive self-compassion have been associated with reduced self-criticism, improved emotional regulation, and greater resilience under performance pressure (15, 16). Despite these promising findings, the specific psychological mechanisms underlying the association between self-compassion and psychological well-being in sport contexts remain insufficiently understood, particularly in non-Western athletic settings (3). This limitation is not merely empirical but conceptual, as psychological constructs may function differently across cultural contexts. In collectivist sport systems, self-related attitudes are often shaped by relational expectations, hierarchical norms, and social evaluation concerns, which may influence how athletes interpret and utilize self-directed psychological resources such as self-compassion. As a result, mechanisms identified in predominantly Western samples may not fully capture the processes that underlie psychological functioning in Korean athletic populations.

### 1.2 Self-compassion and psychological well-being

Self-compassion has emerged as a salient psychological resource for promoting mental health in high-pressure environments such as competitive sports (17). Self-compassion has emerged as a salient psychological resource for promoting mental health in high-pressure environments such as competitive sports (17). By fostering compassionate self-responding and reducing self-critical tendencies (13), this resource buffers individuals against excessive self-criticism, emotional dysregulation, and psychological distress (18). In athletic contexts, self-compassion has been linked to adaptive coping strategies, reduced performance anxiety, and enhanced psychological resilience. Collegiate athletes, who routinely encounter evaluative pressure and public scrutiny, may particularly benefit from self-compassion as a means of preserving emotional balance and intrinsic motivation (17). Regarding these multidimensional outcomes of optimal functioning, empirical evidence suggests that self-compassion is associated with greater self-acceptance, improved emotional regulation, and more adaptive goal pursuit, thereby contributing to various aspects of psychological well-being (19). Athletes who adopt a more compassionate stance toward themselves tend to report higher levels of personal growth, stronger interpersonal relationships, and a clearer sense of purpose (20). Despite these findings, relatively few studies have examined how dimensions of self-compassion contribute to psychological well-being among East Asian collegiate athletes (16). Cultural norms such as collectivism, hierarchical social structures, and tendencies toward self-criticism may shape how self-compassion is expressed and experienced in these populations (21). As a result, the strength and direction of associations between self-compassion and psychological well-being may differ among South Korean athletes compared with Western counterparts (22). This cultural nuance underscores the importance of investigating not only the direct associations between self-compassion and psychological well-being but also the underlying psychological mechanisms—such as resilience and self-efficacy—that may explain these relationships within South Korea's unique sport environment (3).

### 1.3 The mediating role of psychological resilience

Psychological Resilience is broadly defined as the capacity to adapt positively in the face of adversity, enabling individuals to maintain psychological functioning despite exposure to stress (23). In sport settings, resilience helps athletes cope effectively with performance failures, injuries, and the demands of intense competition. It is widely regarded as a protective factor that supports emotional regulation, persistence, and mental health in high-pressure environments such as collegiate athletics (24). Self-compassion is thought to promote resilience by reducing harsh self-judgment and fostering emotional stability following setbacks. Rather than internalizing failure or responding with shame, self-compassionate individuals are more likely to employ adaptive coping strategies and recover more efficiently from adversity (25). In this regard, resilience may serve as an important psychological mechanism through which self-compassion contributes to well-being. Empirical evidence suggests that resilience mediates the association between self-compassion and various psychological outcomes, including emotion regulation, life satisfaction, and mental well-being (20). This mediating process may be particularly salient in East Asian cultural contexts, where persistence, emotional restraint, and endurance are often emphasized. Korean collegiate athletes frequently operate within hierarchical relational structures and demanding training environments that require sustained psychological resilience. In such contexts, self-compassion may play a critical role in cultivating resilience that aligns with cultural expectations while simultaneously supporting mental well-being (26). While some literature conceptualizes resilience primarily as a trait-like moderator that buffers against environmental stressors, the present study posits resilience as a dynamic mediating mechanism. Within the context of athletic challenges, self-compassion functions as an active emotion-regulation strategy that cultivates and replenishes state resilience. From this process-oriented perspective, resilience is not merely a pre-existing baseline characteristic but a developable psychological capacity that serves as a critical bridge between compassionate self-responding and multidimensional psychological well-being.

### 1.4 The moderating role of self-efficacy

Self-efficacy refers to individuals’ beliefs in their ability to successfully execute specific actions and manage challenging situations (27). In competitive sports, self-efficacy is a well-established psychological resource associated with motivation, emotional regulation, and performance outcomes. Athletes with higher levels of self-efficacy tend to persist in the face of adversity, engage in goal-directed behavior, and recover from setbacks with greater confidence (28). Within the framework of the present study, self-efficacy is expected to influence how effectively resilience translates into psychological well-being. From a conditional process perspective, self-efficacy may function as a moderator by shaping the strength and direction of the mediated relationship between self-compassion and psychological well-being (29). Findings from recent research and emerging empirical evidence suggest that self-efficacy can condition the indirect effects of self-compassion through resilience across different well-being domains (30). This moderating role is theoretically grounded in social cognitive theory, which posits that efficacy beliefs regulate how individuals interpret challenges, allocate effort, and sustain coping strategies, thereby influencing whether personal resources such as resilience are mobilized or remain underutilized (27). For example, higher self-efficacy may strengthen the indirect effects of resilience on purpose in life and positive relations with others, whereas in domains such as autonomy and environmental mastery, the mediated effects may weaken as self-efficacy increases. One possible explanation is that individuals with strong efficacy beliefs may rely more on perceived competence than on adaptive coping resources, thereby reducing the functional necessity of resilience in certain domains while amplifying its role in domains that require sustained meaning-making or relational engagement. These patterns indicate that self-efficacy does not uniformly enhance psychological well-being but may exert domain-specific influences depending on the nature of the psychological outcome. Cultural context may further shape how self-efficacy operates within this moderated mediation process. In collectivist settings such as South Korea, self-efficacy may reflect not only perceptions of individual competence but also the internalization of social expectations and hierarchical feedback (31). Under such conditions, efficacy beliefs may function as a regulatory filter that determines when resilience is activated, rather than as a uniformly facilitative resource. As a result, higher self-efficacy may reduce reliance on resilience in certain domains (e.g., autonomy) while enhancing its effects in domains that emphasize interpersonal or value-based components (e.g., purpose in life and positive relations with others) (8). This conditional pattern suggests that self-efficacy operates as a context-sensitive psychological amplifier rather than a linear predictor, strengthening or attenuating the influence of resilience depending on situational and domain-specific demands. These distinctions underscore the importance of conceptualizing self-efficacy not merely as a direct predictor of performance but as a conditional psychological resource that dynamically interacts with other personal strengths.

### 1.5 Purpose of the study and hypotheses

The primary purpose of this study is to examine how dimensions of self-compassion contribute to psychological well-being among collegiate athletes, with particular emphasis on the underlying mechanisms involving resilience and self-efficacy. Given the limited research examining these interrelated processes among Korean collegiate athletes, the present study aims to investigate how dimensions of self-compassion contribute to psychological well-being through resilience and whether this indirect pathway is moderated by self-efficacy. This focus is particularly warranted in the Korean collegiate sport context, where structured training hierarchies, performance-oriented evaluation systems, and dual academic–athletic role demands may intensify psychological strain and modify how personal psychological resources function. Clarifying these mechanisms is essential for informing culturally sensitive interventions that promote mental health and psychological functioning in competitive sport settings. Moreover, identifying culturally grounded mechanisms is practically necessary for developing evidence-based support strategies tailored to Korean athletes rather than relying solely on findings generalized from Western samples. Although previous research has demonstrated associations between self-compassion and various mental health outcomes, the specific processes that explain these relationships remain insufficiently understood—especially within Korean collegiate sport settings, where cultural norms such as collectivism, hierarchical relationships, and performance-oriented expectations may influence how athletes perceive and regulate emotional experiences (32). Understanding these culturally embedded dynamics is essential for identifying how psychological resources operate within unique sociocultural contexts. Building on these theoretical perspectives [including Self-Determination Theory; 33)] and prior empirical findings, this study proposes a conditional process model. First, we hypothesize that resilience functions as a key mediator linking self-compassion dimensions to psychological well-being (34). Athletes who respond to personal challenges with compassion are more likely to exhibit emotional stability and adaptive coping, enhancing their capacity to recover from adversity. However, resilience alone may be insufficient to explain adaptive functioning. Therefore, we further propose that self-efficacy moderates this indirect pathway. Drawing upon Bandura's (27) framework, self-efficacy is defined here as an athlete's internalized belief and psychological assurance in their capacity to successfully execute required sport-specific skills, manage competitive stressors, and achieve desired goals. This conditional relationship suggests that the functional value of resilience may not be uniform across athletes but may vary significantly depending on their perceived competence, ultimately influencing how effectively resilience translates into multidimensional well-being. To evaluate these proposed mechanisms, the present study employed PROCESS Macro Model 14 (30). Conceptually, Model 14 is specifically structured to test second-stage moderated mediation. It estimates the indirect effect of an independent variable on a dependent variable through a mediator, while simultaneously allowing the pathway from the mediator to the dependent variable to be conditioned by a moderating variable. This analytical framework perfectly aligns with our theoretical model, as it provides a structured approach to empirically test whether the bridging role of resilience between self-compassion and well-being is amplified or attenuated by an athlete's self-efficacy.

**Figure 1:**
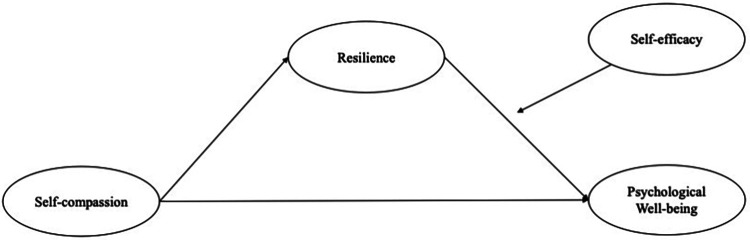
Research model.

Based on the conceptual model (see Figure 1) and prior empirical evidence, the following hypotheses were formulated:

H1. Dimensions of self-compassion will be significantly associated with psychological well-being.
H2. Resilience will mediate the associations between self-compassion dimensions and psychological well-being.
H3. Self-efficacy will moderate the indirect effects of self-compassion dimensions on psychological well-being through resilience (moderated mediation).
H4. The moderated mediation effects will differ across the five domains of psychological well-being (autonomy, environmental mastery, personal growth, purpose in life, and positive relations with others). Collectively, this study aims to clarify the psychological processes through which self-compassion contributes to psychological well-being and to provide culturally grounded insights for enhancing mental health support among Korean collegiate athletes.

## 2 Methods

### 2.1 Participants

A total of 342 collegiate athletes (226 males and 116 females; *M* = 21.18, *SD* = 2.94) voluntarily participated in the study. All participants were officially registered with the Korean Sport & Olympic Committee and represented a wide range of sport disciplines, including individual sports (*n* = 175; e.g., track and field, swimming, taekwondo) and team sports (*n* = 167; e.g., soccer, basketball, volleyball). Athletic experience ranged from 1 to 13 + years. The inclusion criteria were as follows: (a) current enrollment as full-time student-athletes at accredited universities in South Korea; (b) participation in regular collegiate-level training programs supervised by certified coaches; and (c) active competition in official university- or national-level events during the data collection period. The exclusion criteria were as follows: (a) athletes on medical leave or undergoing rehabilitation during the data collection period; and (b) incomplete or duplicate survey submissions. All collected responses met the inclusion criteria, and no cases were excluded from the final analyses. Prior to conducting the main analyses, potential group differences across gender, sport type (individual vs. team), and athletic experience were examined. Independent-sample t-tests and one-way ANOVA revealed no statistically or practically meaningful differences in self-compassion, resilience, self-efficacy, or any dimension of psychological well-being across these demographic variables. Accordingly, these variables were not included as covariates in the final models, consistent with methodological recommendations that covariates should be included only when theoretically justified or empirically associated with the primary study variables (35). The study protocol was approved by the Institutional Review Board of Kyung Hee University, Global Campus [KHGIRB-25-414(EA)]. All participants provided written informed consent in accordance with the Declaration of Helsinki.

### 2.2 Measures

All study variables were assessed using the original self-report instruments that have been widely used and validated in psychological and sport research. As specified below, these instruments were meticulously translated into Korean for the purpose of this study. Participants responded to all items using a 5-point Likert scale ranging from 1 (strongly disagree) to 5 (strongly agree), with higher scores indicating higher levels of the respective constructs. The correlation matrix and descriptive statistics for the key variables are presented in Table 1.

**Table 1:** The correlation between key variables, descriptive statistics and reliability.

Variables	1	2	3	4	5	6	7	8	9	10	11
1. Common humanity	1										
2. Isolation	-.365***	1									
3. Self-judgment	-.265***	.626***	1								
4. Self-kindness	.641***	-.393***	-.358***	1							
5. Autonomy	.558***	-.350***	-.541***	.538***	1						
6. Environmental	.598***	-.374***	-.451***	.479***	.730***	1					
7. Personal growth	.590***	-.373***	-.518***	.513***	.708***	.713***	1				
8. Purpose in life	-.294***	.421***	.759***	-.273***	-.539***	-.494***	-.545***	1			
9. Positive relations with others	.293***	-.238***	-.635***	.281***	.557***	.462***	.616***	-.859***	1		
10. Resilience	.571***	-.207***	.066	.585***	.317***	.386***	.359***	.132*	-.109**	1	
11. Self-efficacy	.588***	-.418***	-.261***	.647***	.536***	.528***	.568***	-.199***	.212***	.760***	1
*M*	4.180	2.085	2.490	4.152	4.020***	4.073	4.021	3.786	3.522	3.745	3.964
*SD*	0.692	0.957	1.149	0.708	0.704	0.639	0.694	1.114	1.229	0.448	0.578
Cronbach Alpha	.817	.873	.888	.839	.674	.639	.632	.845	.901	.852	.858

1–4 are dimensions of self-compassion, 5–9 are dimensions of psychological well-being.

* *p* < .05, ** *p* < .01, *** *p* < .001.

#### 2.2.1 Self-Compassion

Self-compassion was assessed using a 12-item subset of the State Self-Compassion Scale (SCS) (36). As a validated Korean version of this specific 2021 state-level scale was not yet available, the instruments were translated into Korean by the authors following a standard back-translation procedure to ensure linguistic and conceptual equivalence. The study focused on four core dimensions frequently emphasized in sport psychology: self-kindness, common humanity, self-judgment, and isolation, with each subscale consisting of 3 items. A representative item is “I'm giving myself the caring and tenderness I need” (self-kindness). In the present study, the internal consistency (Cronbach's alpha) for the subscales demonstrated good reliability: self-kindness (*α* = .839), common humanity (*α* = .817), self-judgment (*α* = .888), and isolation (*α* = .873).

#### 2.2.2 Resilience

Resilience, defined as the capacity to adapt positively and maintain psychological functioning in the face of stress or adversity, was assessed using the 25-item Connor–Davidson Resilience Scale (CD-RISC) (37). As a formally validated Korean version of this specific scale was not adopted for this study, the instrument was translated into Korean by the authors following a rigorous translation and back-translation procedure to ensure linguistic and conceptual equivalence. A representative item is “I am able to adapt when changes occur.” In the present study, the scale exhibited high internal consistency (Cronbach's *α* = .852).

#### 2.2.3 Self-Efficacy

Self-efficacy was assessed using the 10-item General Self-Efficacy Scale (GSES) developed by Schwarzer and Jerusalem (38). This unidimensional scale measures individuals’ overarching beliefs in their capacity to manage challenging situations and perform desired behaviors. A representative item is “I can always manage to solve difficult problems if I try hard enough.” To ensure linguistic and conceptual equivalence, the instrument was translated into Korean by the authors following a rigorous translation and back-translation procedure. In the present study, the scale demonstrated high internal consistency (Cronbach's *α* = .858).

#### 2.2.4 Psychological well-being

Psychological well-being was assessed using a 15-item subset of the multidimensional scale based on Ryff's (11) model. While the original short-form framework includes six domains, this study focused on five specific domains: autonomy, environmental mastery, personal growth, positive relations with others, and purpose in life, with each subscale consisting of 3 items. A representative item is “I have confidence in my own opinions, even if they are different from the way most other people think” (autonomy). To ensure linguistic and conceptual equivalence, the instrument was translated into Korean by the authors following a rigorous translation and back-translation procedure. Consistent with prior findings that certain psychological well-being dimensions tend to show comparatively lower reliability in East Asian populations (39), the internal consistency coefficients (Cronbach's *α*) in this study were.674 (autonomy),.639 (environmental mastery),.632 (personal growth),.845 (purpose in life), and.901 (positive relations).

## 3 Results

Prior to the main analysis, we verified the fundamental assumptions of multiple regression, including normality, homoscedasticity, and the absence of multicollinearity (all VIFs <10). To investigate the moderated-mediation hypothesis, we conducted a conditional process analysis using Hayes’ (65) Model 14 of the PROCESS macro (version 5.0) with 5,000 bootstrap samples. In the proposed model, a specific dimension of self-compassion was modeled as the independent variable, a dimension of psychological well-being as the dependent variable, resilience as the mediator, and self-efficacy as the moderator of the second stage (the path from mediator to dependent variable). Other dimensions of self-compassion were included as covariates in each analysis to isolate the unique effect of the primary predictor. The conditional indirect effects were evaluated at the mean and ±1 standard deviation (SD) of the moderator. The statistical significance of the moderated mediation was determined by the Index of Moderated Mediation, with 95% bias-corrected confidence intervals (CIs) that do not include zero indicating significance. The analysis results are presented in Table 2.

**Table 2:** Direct and conditional indirect effects of self-compassion on psychological well-being.

Predictor	Effect/Self-Efficacy Level	Dependent Variables (Psychological Well-Being)				
		Autonomy	Environmental Mastery	Personal Growth	Purpose in Life	Positive Relations with Others
Self-Kindness	Direct Effect (SE)	0.10 (0.05)	−0.05 (0.05)	−0.01 (0.05)	0.01 (0.08)	0.10 (0.10)
	Indirect Effect (Low: −1 SD)	0.02 [−0.07, 0.12]	0.09 [0.002, 0.20]	−0.00 [−0.10, 0.10]	0.13 [0.01, 0.27]	−0.22 [−0.42, −0.07]
	Indirect Effect (Mean)	−0.05 [−0.13, 0.02]	0.02 [−0.07, 0.09]	−0.02 [−0.11, 0.06]	0.25 [0.12, 0.43]	−0.34 [−0.53, −0.18]
	Indirect Effect (High: +1 SD)	−0.07 [−0.18, 0.003]	−0.01 [−0.11, 0.08]	−0.03 [−0.14, 0.06]	0.29 [0.14, 0.50]	−0.38 [−0.59, −0.20]
Common Humanity	Direct Effect (SE)	0.29 (0.05)***	0.34 (0.05)***	0.35 (0.05)***	−0.33 (0.07)***	0.42 (0.09)***
	Indirect Effect (Low: −1 SD)	0.01 [−0.04, 0.09]	0.07 [0.002, 0.15]	−0.00 [−0.07, 0.07]	0.09 [0.01, 0.21]	−0.16 [−0.32, −0.04]
	Indirect Effect (Mean)	−0.04 [−0.10, 0.01]	0.01 [−0.05, 0.07]	−0.02 [−0.08, 0.04]	0.18 [0.07, 0.32]	−0.24 [−0.40, −0.11]
	Indirect Effect (High: +1 SD)	−0.05 [−0.13, 0.003]	−0.01 [−0.08, 0.06]	−0.02 [−0.10, 0.05]	0.21 [0.09, 0.36]	−0.27 [−0.44, −0.12]
Self-Judgment	Direct Effect (SE)	−0.26 (0.03)***	−0.18 (0.03)***	−0.25 (0.03)***	0.64 (0.05)***	−0.65 (0.06)***
	Indirect Effect (Low: −1 SD)	0.01 [−0.04, 0.07]	0.05 [0.001, 0.11]	−0.00 [−0.06, 0.06]	0.07 [0.01, 0.15]	−0.13 [−0.24, −0.04]
	Indirect Effect (Mean)	−0.03 [−0.07, 0.01]	0.01 [−0.04, 0.05]	−0.01 [−0.06, 0.03]	0.14 [0.07, 0.23]	−0.19 [−0.30, −0.10]
	Indirect Effect (High: +1 SD)	−0.04 [−0.10, 0.002]	−0.00 [−0.06, 0.04]	−0.02 [−0.08, 0.04]	0.16 [0.08, 0.26]	−0.22 [−0.32, −0.11]
Isolation	Direct Effect (SE)	0.16 (0.04)***	0.06 (0.04)	0.12 (0.04)**	−0.12 (0.05)*	0.42 (0.06)***
	Indirect Effect (Low: −1 SD)	−0.01 [−0.04, 0.02]	−0.03 [−0.07, 0.00]	0.00 [−0.03, 0.03]	−0.04 [−0.10, 0.00]	0.06 [0.01, 0.16]
	Indirect Effect (Mean)	0.01 [−0.00, 0.05]	−0.00 [−0.03, 0.02]	0.01 [−0.02, 0.04]	−0.07 [−0.15, −0.01]	0.10 [0.01, 0.21]
	Indirect Effect (High: +1 SD)	0.02 [−0.00, 0.06]	0.00 [−0.02, 0.04]	0.01 [−0.02, 0.05]	−0.08 [−0.18, −0.01]	0.11 [0.01, 0.23]

Values in square brackets [ ] indicate the 95% bias-corrected bootstrap confidence intervals (CI). Indirect effect refers to the conditional indirect effect of the independent variable on the dependent variable via resilience at different levels of self-efficacy (moderator). CI limits that do not cross zero indicate a significant conditional indirect effect.

* *p* < .05. ** *p* < .01. *** *p* < .001.

The initial stage of the mediation model assessed the impact of self-compassion dimensions on resilience. The overall model was significant, *F*(4, 337) = 91.27, *p* < .001, *R*^2^ = .52. Specifically, common humanity (*b* = 0.21, *p* < .001), self-kindness (*b* = 0.29, *p* < .001), and self-judgment (*b* = 0.17, *p* < .001) all had a significant positive relationship with resilience. Conversely, isolation (*b* = −0.08, *p* < .001) had a significant negative relationship with resilience.

In the moderated mediation analysis for autonomy, the analysis revealed a significant interaction between resilience and self-efficacy (*b* = −0.26, *p* < .001), indicating that the effect of resilience on autonomy is dependent on an individual's level of self-efficacy. The index of moderated mediation was significant for self-kindness [Index = −0.07, 95% CIs (−0.19, −0.00)], common humanity [Index = −0.05, 95% CIs (−0.15, −0.00)], and self-judgment [Index = −0.04, 95% CIs (−0.11, −0.00)]. For these three predictors, the positive indirect effect on autonomy via resilience weakened as self-efficacy increased, becoming non-significant at high levels of self-efficacy. The index of moderated mediation for isolation was not significant [Index = 0.02, 95% CIs (−0.00, 0.07)].

Next, for environmental mastery, there was a significant interaction effect between resilience and self-efficacy (*b* = −0.28, *p* < .001), which indicates that self-efficacy moderates the mediating role of resilience in the path from self-compassion to environmental mastery. The index of moderated mediation was significant for all four dimensions of self-compassion. For self-kindness [Index = −0.08, 95% CIs (−0.20, −0.01)], common humanity [Index = −0.06, 95% CIs (−0.15, −0.01)], and self-judgment [Index = −0.05, 95% CIs (−0.11, −0.01)], the conditional indirect effect on environmental mastery was positive and significant at low levels of self-efficacy but became non-significant as self-efficacy increased. For isolation [Index = 0.02, 95% CIs (0.00, 0.07)], the negative indirect effect on environmental mastery (via resilience) was significant at low levels of self-efficacy and was buffered, becoming non-significant, at higher levels of self-efficacy.

Third, in the model predicting personal growth, the interaction between resilience and self-efficacy was not statistically significant (*b* = −0.09, *p* = .239). Consequently, the indices of moderated mediation were not significant for any of the self-compassion dimensions: self-kindness [Index = −0.03, 95% CIs (−0.14, 0.06)], common humanity [Index = −0.02, 95% CIs (−0.10, 0.04)], self-judgment [Index = −0.01, 95% CIs (−0.08, 0.03)], or isolation [Index = 0.01, 95% CIs (−0.02, 0.05)]. Therefore, the moderated mediation hypothesis was not supported for personal growth. However, the analysis of direct effects revealed significant positive direct effects from common humanity (*b* = 0.35, *p* < .001) and isolation (*b* = 0.12, *p* < .001), and a significant negative direct effect from self-judgment (*b* = −0.25, *p* < .001).

Fourth, for purpose in life, there was a significant interaction effect between resilience and self-efficacy (*b* = 0.45, *p* < .001). This indicates that the strength of the mediated path is dependent on self-efficacy. The index of moderated mediation was significant for all four dimensions. Specifically, for self-kindness [Index = 0.13, 95% CIs (0.04, 0.27)], common humanity [Index = 0.09, 95% CIs (0.03, 0.19)], and self-judgment [Index = 0.07, 95% CIs (0.02, 0.14)], the positive indirect effect via resilience became stronger as self-efficacy increased. For isolation [Index = −0.04, 95% CIs (−0.09, −0.00)], the negative indirect effect via resilience also became stronger (more negative) at higher levels of self-efficacy.Finally, for positive relations with others, a significant interaction effect between resilience and self-efficacy was found (*b* = −0.44, *p* < .001). The index of moderated mediation was significant for self-kindness [Index = −0.13, 95% CIs (−0.27, −0.01)], common humanity [Index = −0.09, 95% CIs (−0.19, −0.00)], and self-judgment [Index = −0.07, 95% CIs (−0.14, −0.00)]. For these predictors, the indirect effect via resilience was negative and became stronger (more negative) as self-efficacy increased. The index for isolation was not significant [Index = 0.04, 95% CIs (−0.00, 0.10)].

## 4 Discussion

The present study examined how dimensions of self-compassion are associated with psychological well-being among Korean collegiate athletes and whether these associations operate through resilience, contingent on levels of self-efficacy. Using a moderated mediation framework (PROCESS Model 14), the findings demonstrated that resilience served as a significant psychological mechanism linking multiple dimensions of self-compassion to psychological well-being. Moreover, self-efficacy functioned as a conditional factor that shaped the strength and direction of these indirect effects across different well-being domains. Collectively, the results extend prior research by elucidating the domain-specific and culturally embedded processes through which self-compassion contributes to athletes’ psychological functioning.

### 4.1 Self-compassion, resilience, and psychological well-being

Consistent with theoretical perspectives emphasizing self-compassion as an adaptive emotional resource (13), the present findings indicate that resilience mediates the relationship between self-compassion dimensions and psychological well-being across several domains (20). Athletes who respond to personal difficulties with greater self-kindness and a sense of shared humanity, while experiencing lower levels of self-judgment and isolation, appear better equipped to recover from adversity and maintain psychological functioning (40). This pattern supports the notion that self-compassion fosters resilience by reducing maladaptive self-evaluative responses and promoting emotional stability following setbacks (41). It is important to note an unexpected statistical pattern regarding the self-judgment dimension. While our theoretical framework suggests that lower self-judgment is adaptive, the multiple regression within the mediation model revealed a significant positive association between self-judgment and resilience. This discrepancy is highly indicative of a statistical suppression effect. Because the dimensions of self-compassion are intercorrelated, entering them simultaneously into the model partials out their shared variance. Consequently, the remaining unique variance of self-judgment exhibited a reversed, positive relationship with resilience. Such suppression effects are common when using multidimensional scales with high internal overlap. Therefore, while the global theoretical premise holds true—that reducing maladaptive self-evaluation fosters resilience—the isolated beta weight of self-judgment in this simultaneous model reflects a statistical artifact of controlling for other self-compassion dimensions rather than a standalone conceptual benefit of self-judgment.

These findings strongly align with recent literature from 2020 to 2025 that reconceptualizes how psychological resources operate in athletic contexts. For instance, a recent scoping review by Cormier et al. (16) highlighted self-compassion as a critical facilitator of adaptive emotional regulation in sport, moving beyond mere stress reduction. Furthermore, our conceptualization of resilience as an active mediating process is supported by Gucciardi et al. (34), who posited that resilience is best understood as a dynamic recovery trajectory following adversity rather than a static trait. Additionally, recent empirical evidence indicates that adaptive emotion regulation effectively mitigates athletic burnout and sustains psychological well-being (2). In light of these recent studies, our results substantiate the notion that self-compassion actively fuels this dynamic resilience process, thereby functioning as a vital mechanism for psychological preservation in high-pressure collegiate environments. In competitive sport environments characterized by frequent evaluation and performance pressure, such adaptive responses may be particularly critical for sustaining well-being (10). From a regulatory perspective, self-compassion may facilitate adaptive functioning not only through resilience but also through alternative psychological pathways, such as cognitive reappraisal, acceptance, and reduced rumination, which can independently support well-being even when resilience is not the primary mechanism. Importantly, the mediating role of resilience was not uniform across all dimensions of psychological well-being. While resilience significantly mediated associations for autonomy, environmental mastery, purpose in life, and positive relations with others, no significant conditional indirect effect was observed for personal growth (42). This finding suggests that personal growth may be influenced by self-compassion through more direct or alternative pathways that are less dependent on resilience (42). One theoretical explanation is that personal growth represents a developmental and meaning-oriented construct that is shaped by reflective self-processing rather than immediate stress-recovery mechanisms.

In developmental terms, growth-oriented outcomes may reflect longer-term meaning-making processes rather than immediate adaptive responses to stress, which could explain the absence of a resilience-based indirect effect in this domain (43).

In this sense, resilience may function primarily as a short-term adaptive resource, whereas personal growth may depend more strongly on longitudinal processes such as identity integration, value clarification, and self-concept reconstruction. Cultural context may further contribute to this pattern. In collectivist environments such as South Korea, personal growth is often construed in relational or socially embedded terms rather than purely individual development, which may weaken the role of resilience as an intermediary mechanism while strengthening the influence of reflective or socially mediated processes.

### 4.2 The conditional role of self-efficacy

A key contribution of this study lies in demonstrating that self-efficacy does not uniformly enhance the beneficial effects of resilience but instead exerts domain-specific influences (44). Higher self-efficacy strengthened the indirect effects of resilience on purpose in life and positive relations with others, suggesting that confidence in one's abilities may amplify value-based and interpersonal aspects of well-being (45). In contrast, the mediated effects weakened for autonomy and environmental mastery as self-efficacy increased (46). One possible interpretation is that athletes with strong beliefs in their competence may rely less on resilience-related processes when navigating domains that emphasize personal control or independence (47). More specifically, individuals with high self-efficacy may approach autonomy- and mastery-related situations through direct agentic regulation rather than adaptive recovery mechanisms, thereby reducing the functional role of resilience in these domains.

In such cases, perceived competence may act as a psychological substitute for resilience, allowing athletes to manage demands proactively instead of reactively coping with adversity.Specifically, whereas resilience functions primarily as a reactive recovery mechanism triggered after encountering a stressor or failure, high perceived competence operates proactively. Athletes with strong self-efficacy are more likely to appraise potential threats as manageable challenges rather than overwhelming obstacles. Consequently, their robust confidence buffers the initial psychological impact of the stressor, effectively pre-empting the distress and reducing the necessity to activate secondary recovery mechanisms (i.e., resilience) to maintain their sense of autonomy and environmental mastery. This domain-specific moderation is highly consistent with recent advancements in motivation and sports psychology research. For example, recent developments in Self-Determination Theory (48) emphasize that perceived competence (self-efficacy) must be properly balanced with autonomy to fully satisfy psychological needs; thus, high self-efficacy alone may not uniformly optimize all dimensions of well-being. Moreover, recent investigations into the psychological and emotional aspects of student-athletes (1) suggest that personal resources often interact in complex, non-linear ways depending on the specific athletic demands. Within the collectivist performance environments typical of South Korea, recent culturally nuanced assessments (49) further reveal that athletes’ efficacy beliefs are heavily intertwined with hierarchical expectations. These nuanced patterns highlight the importance of conceptualizing self-efficacy as a conditional psychological resource rather than a universally beneficial factor (50). In line with conditional process models, the present findings suggest that the adaptive value of resilience depends on athletes’ perceived competence and the specific psychological outcome under consideration (51). This interpretation is also consistent with regulatory control theories suggesting that when internal control beliefs are already strong, the incremental contribution of secondary adaptive resources becomes attenuated. Such domain-specific effects underscore the limitations of treating psychological resources as uniformly additive and point to the need for more differentiated theoretical models in sport psychology (24). From a cultural standpoint, this pattern may be particularly pronounced in structured sport environments such as those commonly observed in South Korea, where athletes with high perceived competence are often granted greater autonomy and decision-making latitude, potentially diminishing the necessity of resilience as an intermediary mechanism.

### 4.3 Cultural context and theoretical implications

The observed patterns can be better understood within the distinct cultural context of South Korean collegiate sport (49). In collectivist and highly structured environments characterized by clear authority gradients, self-efficacy often reflects both individual confidence and the internalization of external validation from coaches and performance evaluations (52). Consequently, athletes’ perceived competence is deeply intertwined with social expectations. Within such hierarchical climates, high self-efficacy may reduce reliance on resilience in domains emphasizing autonomy, while simultaneously enhancing its benefits in domains involving relational meaning or life purpose (53). This suggests that psychological resources do not operate merely as independent, additive factors; rather, they function as contextually contingent mechanisms whose roles shift based on structural expectations and interpersonal dynamics.

From a theoretical standpoint, particularly within Self-Determination Theory (48), these findings demonstrate that self-compassion, resilience, and self-efficacy interact differently in fulfilling basic psychological needs depending on the cultural context (54). Furthermore, by adopting a dimensional approach to self-compassion and examining moderated mediation across multiple well-being domains, the present study advances existing literature that traditionally relies on global constructs and uniform effect assumptions (36). Ultimately, interpreting these findings through the lens of Korean sport culture strengthens theoretical coherence and enhances ecological validity, highlighting the necessity of considering domain specificity and sociocultural systems when examining athletes’ psychological well-being (55).

### 4.4 Practical implications

The findings of the present study offer several practical implications for mental health promotion and coaching practice in collegiate sport settings (9). Interventions aimed at enhancing athletes’ well-being may benefit from simultaneously fostering self-compassion and resilience rather than focusing solely on performance confidence (15). Training programs that encourage compassionate responses to mistakes and setbacks while reducing self-critical tendencies may help athletes develop adaptive coping capacities that support psychological functioning under pressure (20). Coaches may therefore benefit from recognizing self-compassion as a foundational psychological resource that supports well-being beyond performance outcomes (56). The mediating role of resilience highlights the importance of integrating resilience-building strategies into daily training environments (57). Coaches can facilitate resilience by normalizing failure as part of the learning process, providing constructive feedback, and fostering psychologically safe climates in which athletes feel supported when facing adversity (58). Such practices may be particularly effective in promoting autonomy, environmental mastery, purpose in life, and positive interpersonal relationships among athletes (59). Importantly, the domain-specific moderating effects of self-efficacy indicate that confidence-building interventions should be applied with nuance rather than assumed to be uniformly beneficial (60). While enhancing self-efficacy may strengthen value-based and relational aspects of well-being, such as purpose in life and positive relations, excessive emphasis on individual competence may inadvertently weaken adaptive processes related to autonomy or environmental mastery (61). Accordingly, practitioners should carefully integrate confidence-enhancement approaches with resilience-oriented strategies depending on targeted psychological outcomes (62). Finally, within the cultural context of Korean collegiate sport, coaching practices that align self-compassion and resilience training with relational sensitivity and hierarchical structures may be particularly effective (26). Coaches who model compassionate responses, acknowledge effort alongside outcomes, and emphasize collective growth may enhance athletes’ psychological well-being while maintaining performance standards (63). Building upon these empirical findings, sport psychology professionals and athletic departments should develop structured enhancement and intervention programs tailored specifically for collegiate athletes. Such comprehensive programs could practically integrate self-compassion workshops to build psychological resilience, alongside mastery-oriented goal-setting sessions designed to boost self-efficacy. By simultaneously targeting both resilience and self-efficacy through evidence-based interventions, practitioners can more effectively buffer athletes against the dual stressors of academic and athletic demands, ultimately fostering a more robust and multidimensional psychological well-being. Collectively, these findings underscore the value of adopting psychologically informed coaching approaches that integrate self-compassion, resilience, and self-efficacy in a context-sensitive manner (64).

### 4.5 Limitations and future directions

Several limitations should be acknowledged. First, the cross-sectional design precludes causal inferences regarding the observed relationships. Longitudinal or experimental studies are needed to establish temporal ordering and causal mechanisms. Second, reliance on self-report measures may introduce response bias, although validated instruments were employed. Future research could incorporate behavioral or physiological indicators of resilience and well-being. Additionally, future studies should investigate why demographic factors such as athletic experience did not significantly differentiate psychological outcomes in this study. It is plausible that severe selection biases in elite sports, combined with the overriding pressures of current collegiate environments, homogenize these psychological traits, highlighting the need to examine athletes’ psychological development longitudinally rather than solely relying on cumulative years of experience. Finally, although the sample provides valuable insight into Korean collegiate athletes, caution is warranted in generalizing the findings to other cultural or athletic contexts. Comparative cross-cultural studies would further clarify the generalizability of the proposed model.

### 4.6 Conclusion

In conclusion, the present study elucidates the multidimensional and conditional processes through which self-compassion contributes to psychological well-being among collegiate athletes. By identifying resilience as a key mediating mechanism and self-efficacy as a domain-specific moderator, the findings highlight the complex interplay of psychological resources in shaping athletes’ mental health. These insights contribute to a more nuanced understanding of well-being in competitive sport and offer a foundation for developing culturally sensitive and theoretically informed interventions.

## Data Availability

The original contributions presented in the study are included in the article/Supplementary Material, further inquiries can be directed to the corresponding author.
